# Synthesis, crystal structure and properties of *catena*-poly[[bis­(4-methyl­pyridine-κ*N*)cobalt(II)]-di-μ-thio­cyanato-κ^2^*N*:*S*;κ^2^*S*:*N*], which shows a rare coordination geometry

**DOI:** 10.1107/S2056989024012003

**Published:** 2025-01-01

**Authors:** Christian Näther, Jan Boeckmann

**Affiliations:** a Institut für Anorganische Chemie, Universität Kiel, Max-Eyth.-Str. 2, 24118 Kiel, Germany; University of Hyogo, Japan

**Keywords:** synthesis, crystal structure, coordination polymer, chain structure, spectroscopic properties, cobalt thio­cyanate, 4-methyl­pyridine

## Abstract

Reaction of Co(NCS)_2_ with 4-methyl­pyridine in water leads to the formation of single crystals of the title compound, [Co(NCS)_2_(C_6_H_7_N)_2_]_*n*_. The asymmetric unit consists of two crystallographically independent thio­cyanate anions and two crystallographically independent 4-methyl­pyridine coligands in general positions, as well as of two different Co^II^ cations, of which one is located on a twofold rotational axis, whereas the second occupies a center of inversion. The methyl H atoms in both 4-methyl­pyridine ligands are disordered and were refined using a split model. Both Co^II^ cations are octa­hedrally coordinated by two N- and two S-bonded thio­cyanate anions and two 4-methyl­pyridine coligands and are linked by pairs of 1,3-bridging anionic ligands into chains. Within these chains the cations show an alternating all-*trans* and *cis*–*cis*–*trans* configuration, which leads to the formation of corrugated chains. Powder X-ray diffraction proves that a pure crystalline phase has been obtained and the values of the CN stretching vibrations of the anionic ligands observed in the IR and the Raman spectra are in agreement with the presence of bridging anionic ligands.

## Abstract 2

In the crystal structure of the title compound, Co(NCS)_2_(C_6_H_7_N)_2_ (C_6_H_7_N = 4-methyl­pyridine), the Co^II^ cations are in an alternating octa­hedral all-*trans* and *cis*-*cis*-*trans* coordination and linked into corrugated chains by pairs of μ-1,3 bridging thio­cyanate anions.

## 1. Chemical context

For a long time, our inter­est has focused on the synthesis and crystal structure of transition-metal thio­cyanate coordination compounds based on Mn^II^, Fe^II^, Co^II^ and Ni^II^, because they show a large structural variability, which can partly be traced back to the versatile coordination behavior of this anionic ligand (Näther *et al.*, 2013 ▸). In nearly all cases these cations are in an octa­hedral coordination, even though with cobalt several compounds with a tetra­hedral coordination are also known. Within this project we are especially inter­ested in compounds in which the metal cations are linked into chains or layers, because such compounds show versatile magnetic behavior (Neumann *et al.*, 2018 ▸; Suckert *et al.*, 2016 ▸). This is especially the case for compounds based on cobalt, which can show 1D or 3D ferromagnetic ordering (Mautner *et al.*, 2018 ▸; Jochim *et al.*, 2020 ▸; Rams *et al.*, 2017*a* ▸, 2020 ▸).

In most cases, chain compounds are observed in which the metal cations are in an octa­hedral all-*trans* coordination, leading to the formation of linear chains. Linear chains are also observed for a *cis–cis–trans*-coordination if the neutral coligands are in *trans*-positions, which is the case, for example, in *M*(NCS)_2_(4-benzoyl­pyridine)_2_ with *M* = Co, Ni [refcodes ODEYII (Rams *et al.*, 2017*b* ▸) and GIQQUV (Jochim *et al.*, 2018 ▸)] or in Co(NCS)_2_(2,3-di­methyl­pyrazine-1,4-dioxide) (PEVZOG; Shi *et al.*, 2007 ▸). Corrugated chains are observed if the two bridging S-bonded thio­cyanate anions are in an *trans*-position like in Mn(NCS)_2_(4-nitro­pyridine *N*-oxide (SINKUW; Shi *et al.*, 2006*a* ▸) or in Ni(NCS)_2_(2,2′-bi­pyridine (GIQREG; Jochim *et al.*, 2018 ▸). If the two bridging N-bonded thio­canate anions are in a *trans*-position like in Ni(NCS)_2_[1-(2-amino­eth­yl)pyrrolidine-*N*,*N*′) (ABOBIC; Maji *et al.*, 2001 ▸) corrugated chains are also observed, Finally, in Ni(NCS)_2_(4-methyl­pyridine *N*-oxide [PEDSUN (Shi *et al.*, 2006*b* ▸) and PEDSUN0 (Marsh, 2009 ▸)] an all-*cis* configuration is observed that also leads to the formation of corrugated chains.

In this context it is noted that we have reported on Co and Ni compounds with the composition Ni(NCS)_2_(4-chloro­pyridine)_2_ (UHUVIF and UHUVIF01; Jochim *et al.*, 2018 ▸ and Co(NCS)_2_(4-chloro­pyridine)_2_ (GIQQIJ and GIQQIJ01; Böhme *et al.*, 2020 ▸) for each of which two isomers exist. In one of these isomers the metal cations are in an all-*trans* configuration, whereas in the second isomer that is thermodynamically stable at room temperature, an alternating all-*trans* and *cis*–*cis*–*trans* configuration is observed. Based on these results, we tried to prepare compounds with Ni(NCS)_2_ and 4-methyl­pyridine as ligand for which, because of the chloro–methyl exchange rule (Desiraju & Sarma, 1986 ▸), similar structures can be expected, but only one isomer with the composition Ni(NCS)_2_(4-methyl­pyridine)_2_ was obtained, which is isotypic to the stable isomer of Ni(NCS)_2_(4-chloro­pyridine)_2_ with an alternating all-*trans* and *cis*–*cis*–*trans* configuration (Näther & Mangelsen, 2024 ▸).

In the course of our systematic work we became inter­ested in Co(NCS)_2_ compounds with 4-methyl­pyridine as coligand, to check which of the two isomers might form and if this compound is isotypic to the corresponding Ni compound. It is noted that some of such compounds are already reported with this ligand. Most of them consist of solvates of discrete complexes but one chain compound is reported, for which no atomic coordinates are presented (see *Database survey*).
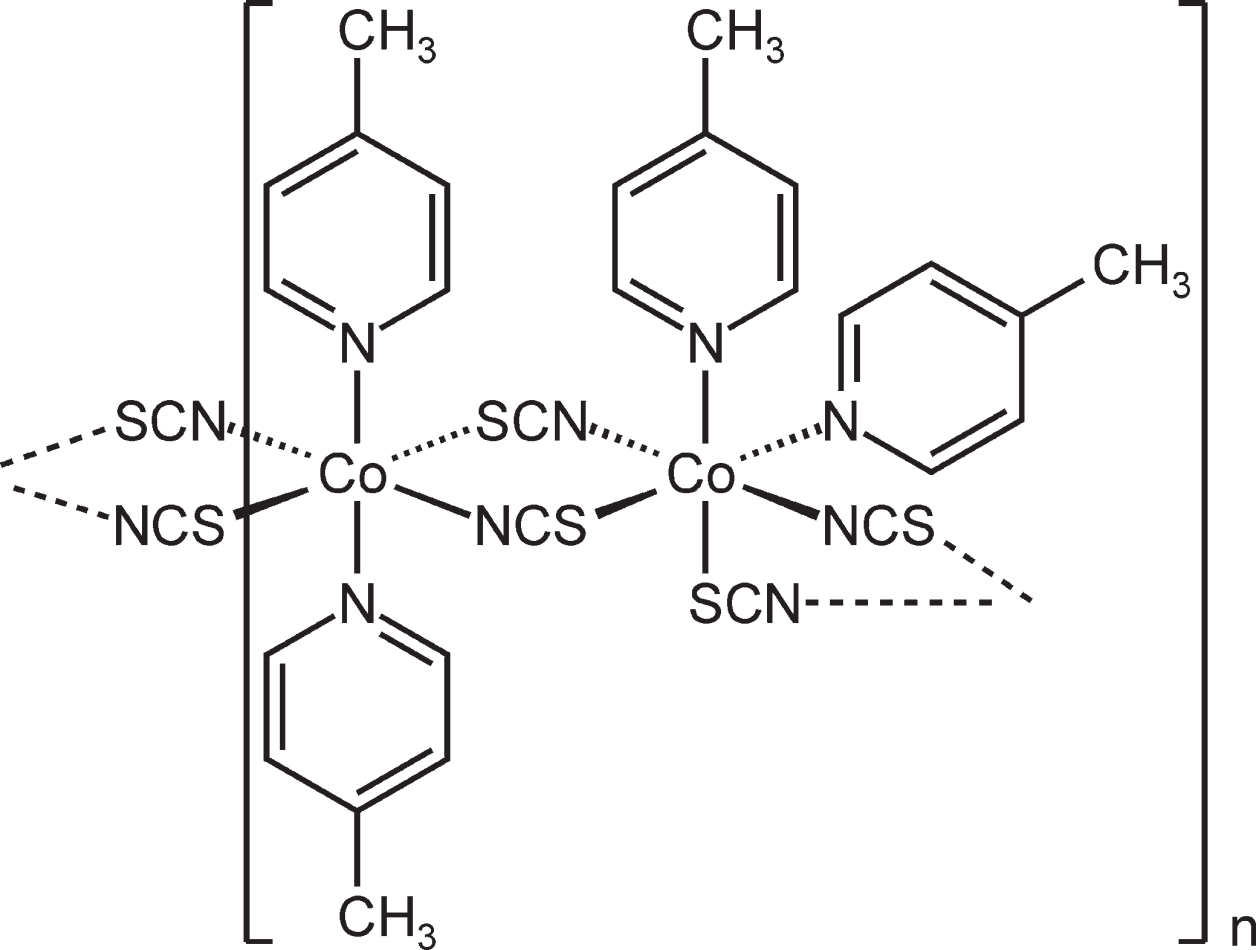


## 2. Structural commentary

**Figure 1:**
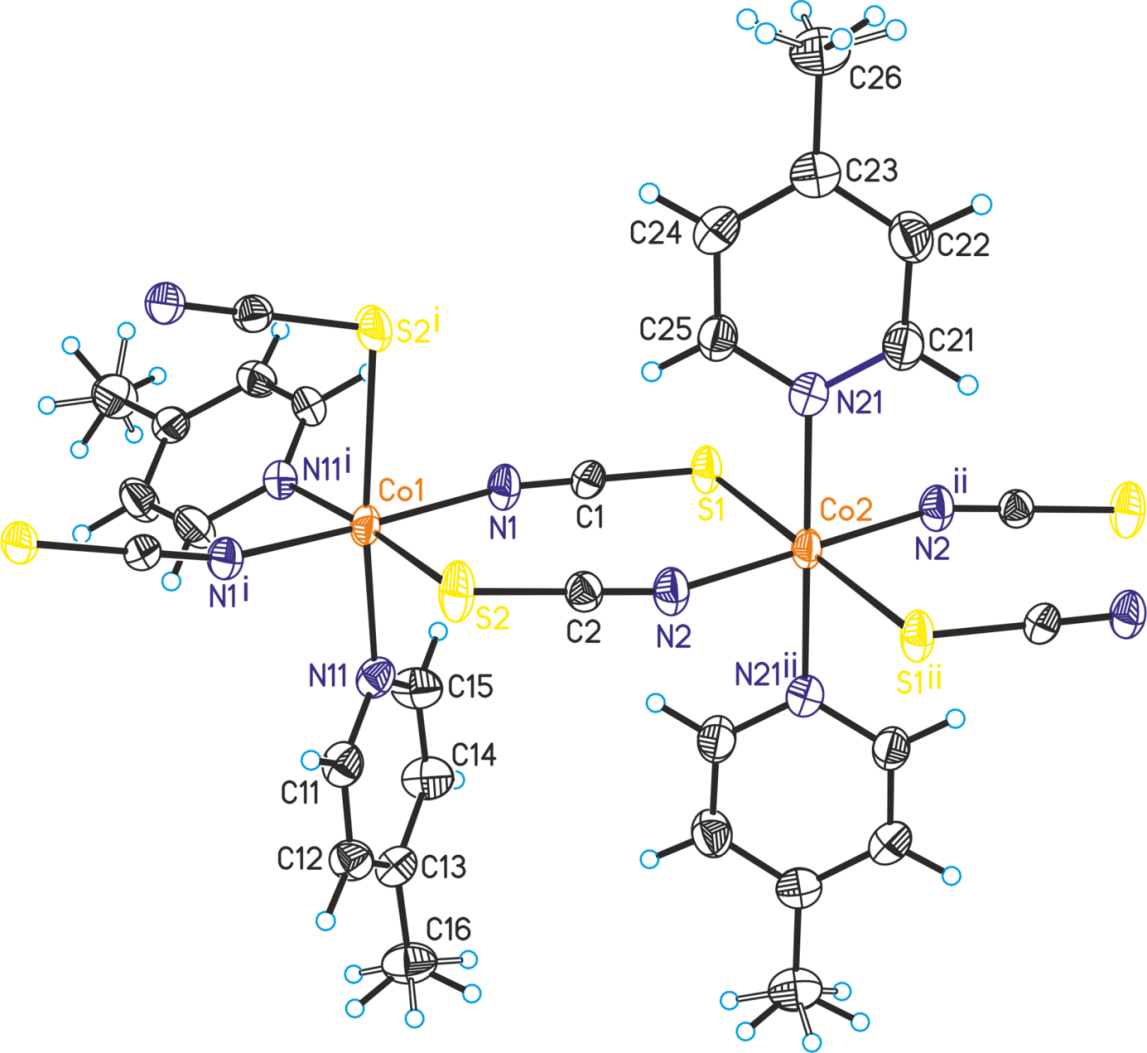
Crystal structure of the title compound with labeling and displacement ellipsoids drawn at the 50% probability level. Symmetry codes: (i) −*x* + 1, *y*, −*z* + 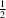; (ii) −*x* + 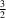, −*y* + 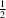, −*z* + 1. The disorder of the methyl H atoms is shown with full and open bonds.

**Figure 2:**
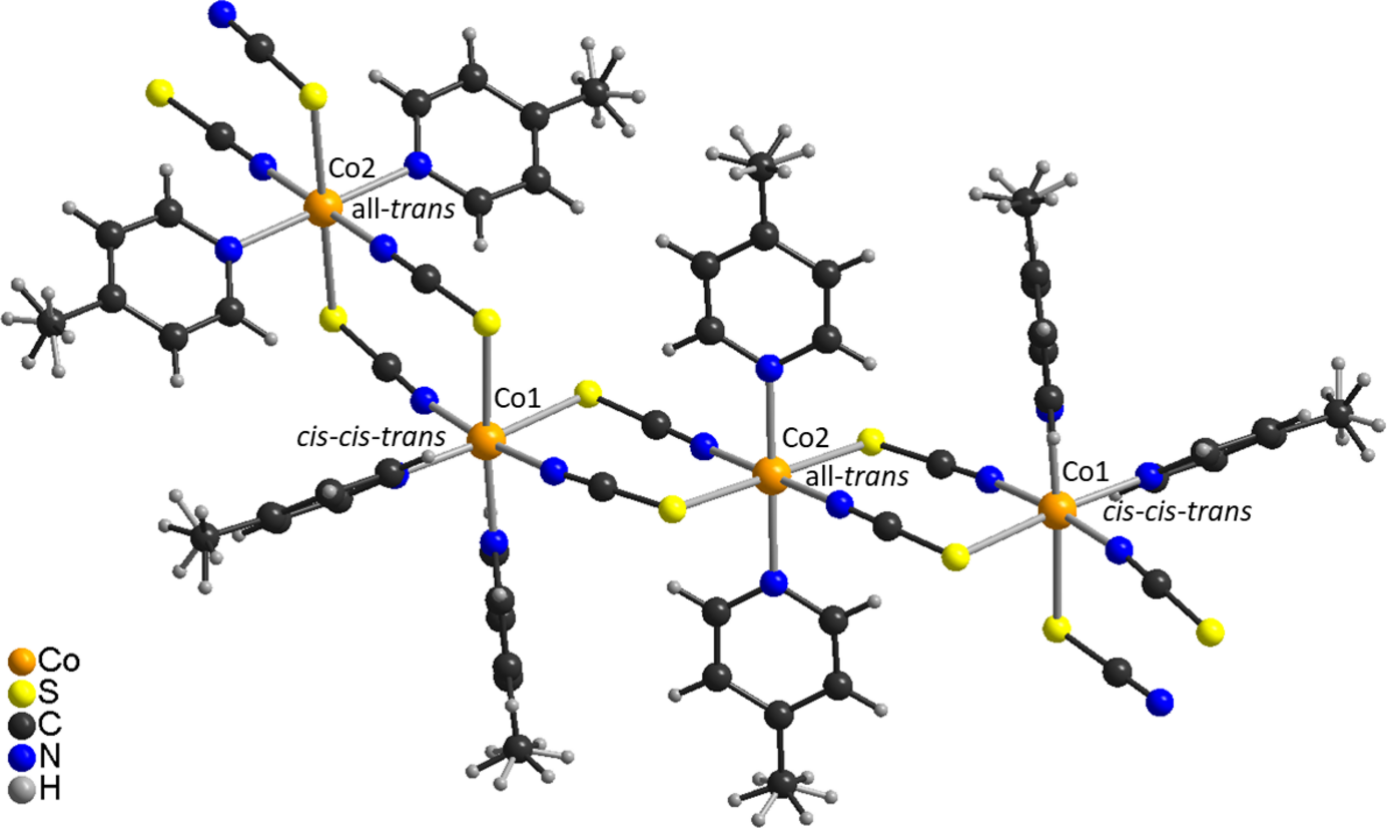
Crystal structure of the title compound with view of a part of a chain with labeling of the Co^II^ cations and showing the actual metal configuration. For clarity the disorder of the methyl H atoms is not shown.

**Figure 3:**
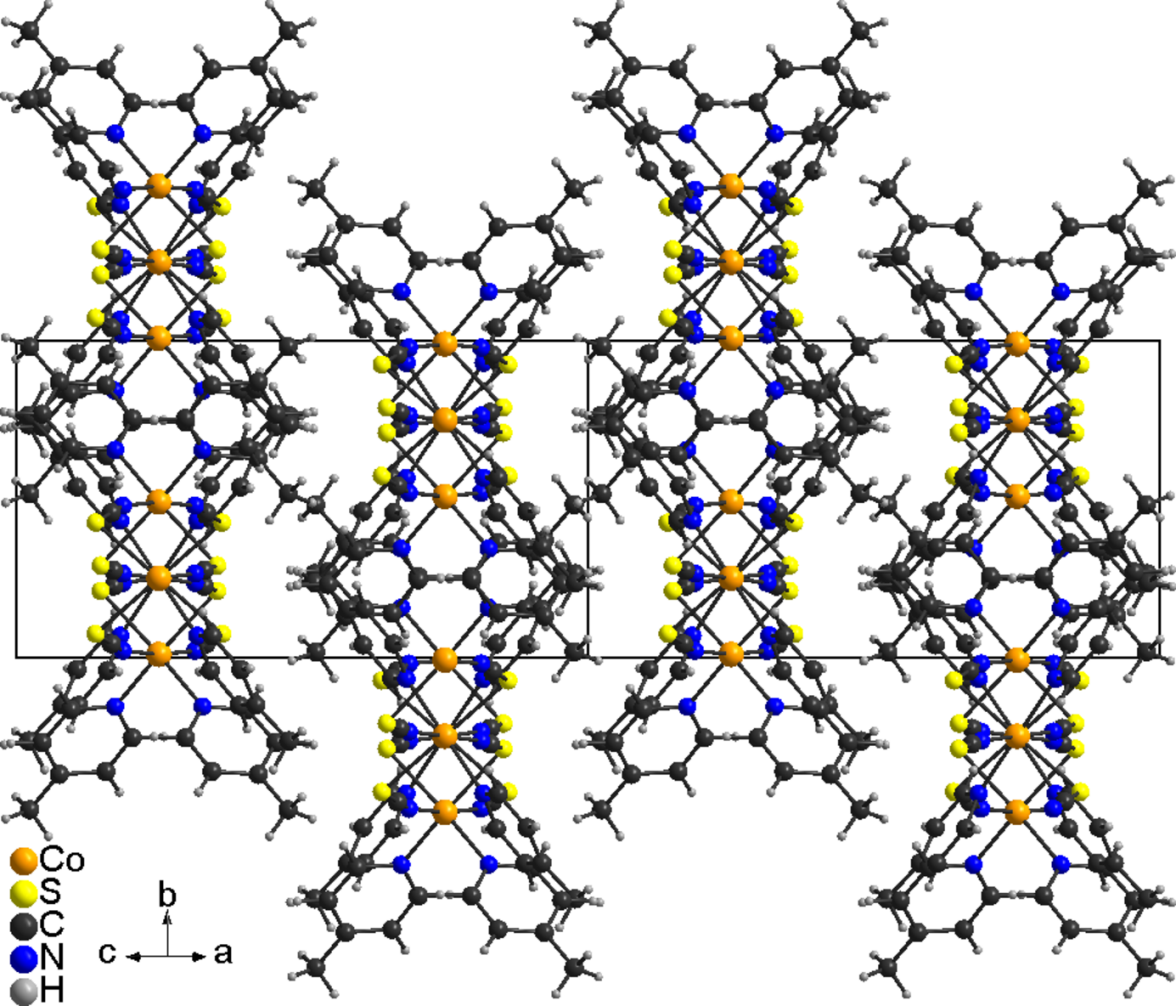
Crystal structure of the title compound in a view along [101]. For clarity the disorder of the methyl H atoms is not shown.

The asymmetric unit of the title compound, Co(NCS)_2_(C_6_H_7_N)_2_, is built up of two crystallographically independent thio­cyanate anions and two crystallographically independent 4-methyl­pyridine coligands in general positions, as well as of two crystallographically independent Co^II^ cations, of which one is located on a twofold rotational axis whereas the second occupies a center of inversion (Figs. 1 ▸ and 2 ▸). The methyl H atoms in both 4-methyl­pyridine ligands are disordered and were refined in two different orientations. Both Co^II^ cations are octa­hedrally coordinated by two 4-methyl­pyridine ligands and two N- and two S-bonding thio­cyanate anions (Figs. 1 ▸ and 2 ▸). One of the Co^II^ cations (Co1) shows a *cis*–*cis–trans* configuration with the thio­cyanate N atoms in a *trans* position and the pyridine N atom as well as the thio­cyanate S atom in *cis* positions (Fig. 2 ▸). The second crystallographically independent Co^II^ cation (Co2) shows an all-*trans* configuration (Fig. 2 ▸). For the Co^II^ cation that shows a *cis*–*cis–trans* configuration, the Co—N distances to the 4-methyl­pyridine ligands are slightly shorter compared to the cation in the *cis–cis–trans* configuration (Table 1 ▸). Moreover, from the bond lengths and angles it is obvious that the octa­hedra are slightly distorted. The metal cations are linked by pairs of μ-1,3-bridging thio­cyanate anions into chains that, because of the alternating all-*trans* and *cis*–*cis*–*trans* configurations, are corrugated (Fig. 3 ▸). It is noted that the title compound is isotypic to the corresponding compound with Ni(NCS)_2_ (Näther & Mangelsen, 2024 ▸) and to the isomer that is thermodynamically stable at room temperature of Ni(NCS)_2_(4-chloro­pyridine)_2_, which proves that the chloro–methyl exchange rule is valid in this case. From our synthetic work there is no hint of the existence of a second isomer of the title compound as observed for the corresponding 4-chloro­pyridine compound. Finally, it is noted that the title compound with an alternating all-*trans* and *cis*–*cis*–*trans* configuration shows a very rare Co coordination.

## 3. Supra­molecular features

In the crystal structure of the title compound, the chains elongate along [101] with each chain surrounded by six neighboring chains (Fig. 3 ▸). There are no significant inter­molecular C—H⋯N or C—H⋯S contacts and there are also no hints of any π–π stacking inter­actions (Table 2 ▸).

## 4. Database survey

A search in the CSD (version 5.43, last update December 2024; Groom *et al.*, 2016 ▸) using CONQUEST (Bruno *et al.*, 2002 ▸) for compounds based on Co(NCS)_2_ and 4-methyl­pyridine revealed that some such compounds are already reported. This includes a compound with the composition Co(NCS)_2_(4-methyl­pyridine)4·*p*-xylene in which the Co cations are tetra­hedrally coordinated by only one N-bonding thio­cyanate anion and three 4-methyl­pyridine ligands and which crystallizes with additional *p*-xylene solvate mol­ecules (Refcode: QQQGKJ; Solaculu *et al.*, 1974 ▸). However, no atomic coordinates are given and no charge balance is achieved, which means that the existence of this compound is questionable. There is also one compound with the composition Co(NCS)_2_(4-methyl­pyridine)_2_bis­(*p*-toluidine)_2_ reported for which also no atomic coordinates are given (Refcode: CECDAP; Micu-Semeniuc *et al.*, 1983 ▸). Surprisingly, the unit-cell parameters are very similar and the crystal system identical to that of compounds built up of octa­hedral discrete complexes with additional solvate mol­ecules (see below).

All remaining compounds consists of discrete complexes with the composition Co(NCS)_2_(4-methyl­pyridine)_4_ that crystallize as clathrates with *p*-toluidine (Refcode CECCOC; Micu-Semeniuc *et al.*, 1983 ▸), 4-methyl­pyridine [Refcodes: XIHHEB (Harris *et al.*, 2001 ▸) and XIHHEB01 (Harris *et al.*, 2003 ▸)] and nitro­benzene (Refcode ZZZUXU), nitro­ethane (Refcode: ZZZUXY) and benzene solvate (Refcode: ZZZUYI; Belitskus *et al.*, 1963 ▸). However, only for one of these compounds (XIHHEB) are atomic coordinates available. Finally, the crystal structure of the pure complex Co(NCS)_2_(4-methyl­pyridine)_4_ is also reported but the unit-cell parameters are identical to that of several clathrates, which indicates that the solvent was not located (Refcode: VERNUC; Harris *et al.*, 2003 ▸).

## 5. Additional investigations

**Figure 4:**
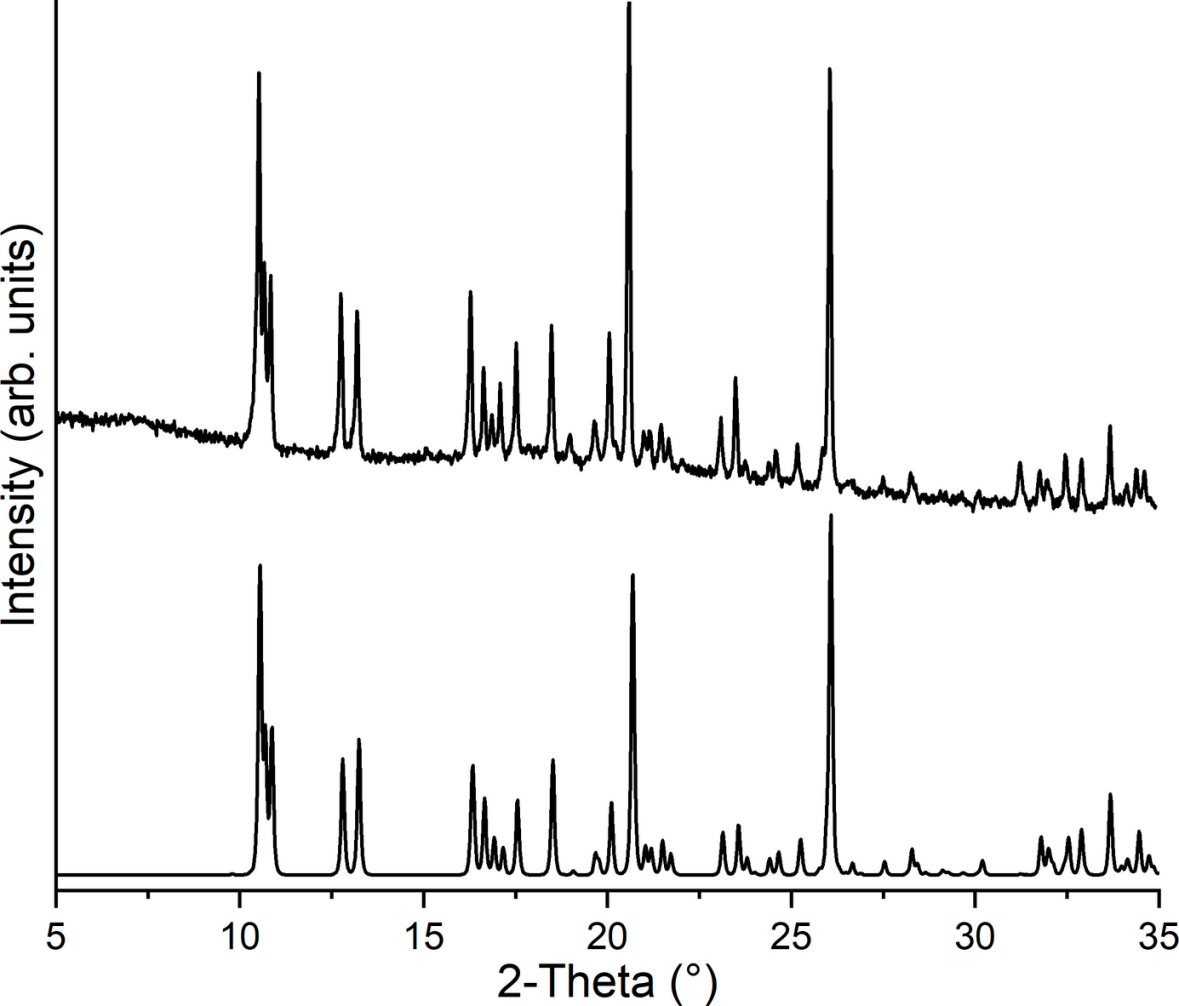
Experimental (top) and calculated (bottom) X-ray powder pattern for the title compound.

**Figure 5:**
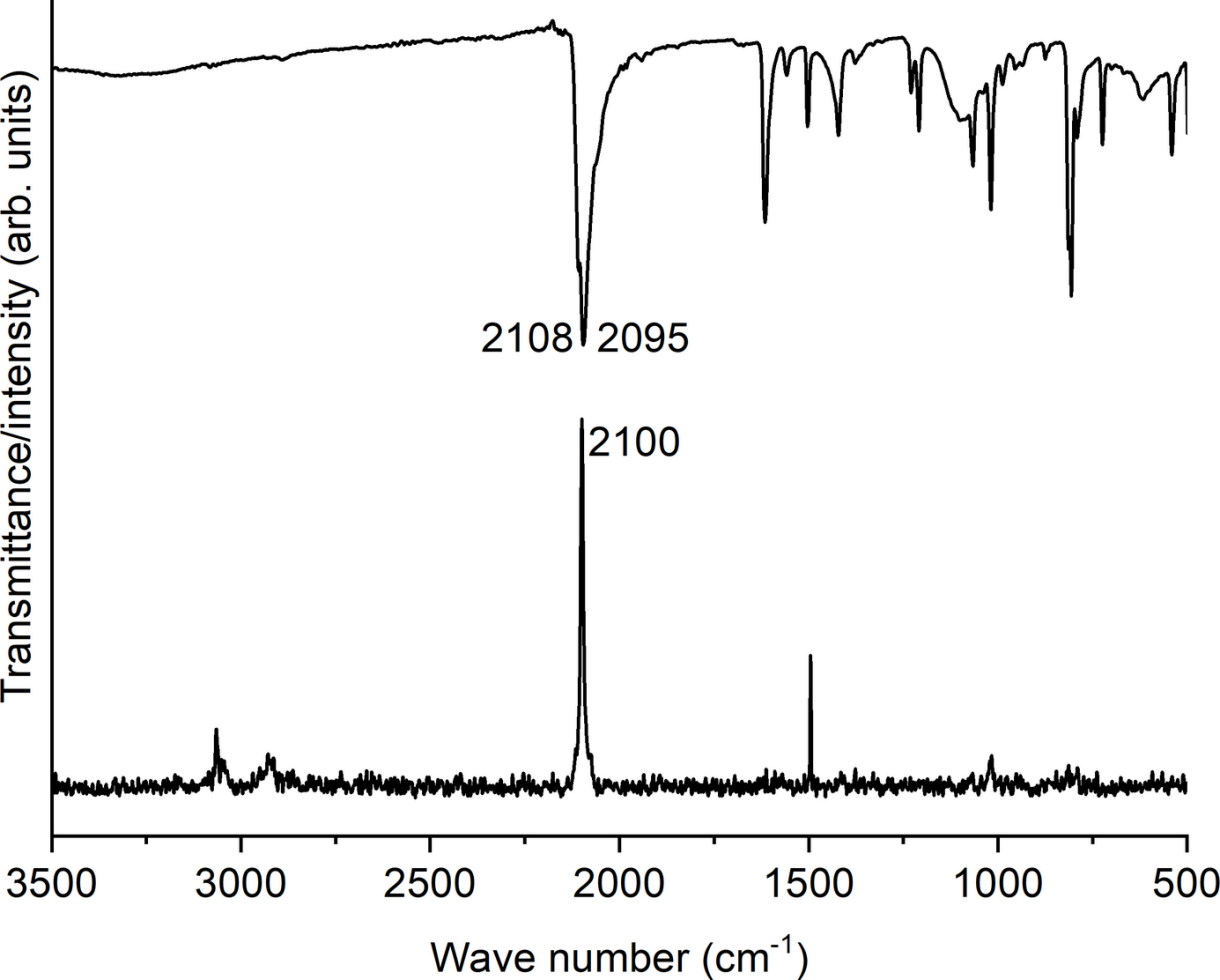
IR (top) and Raman (bottom) spectra of the title compound. The CN stretching vibration of the thio­cyanate anions is given.

**Figure 6:**
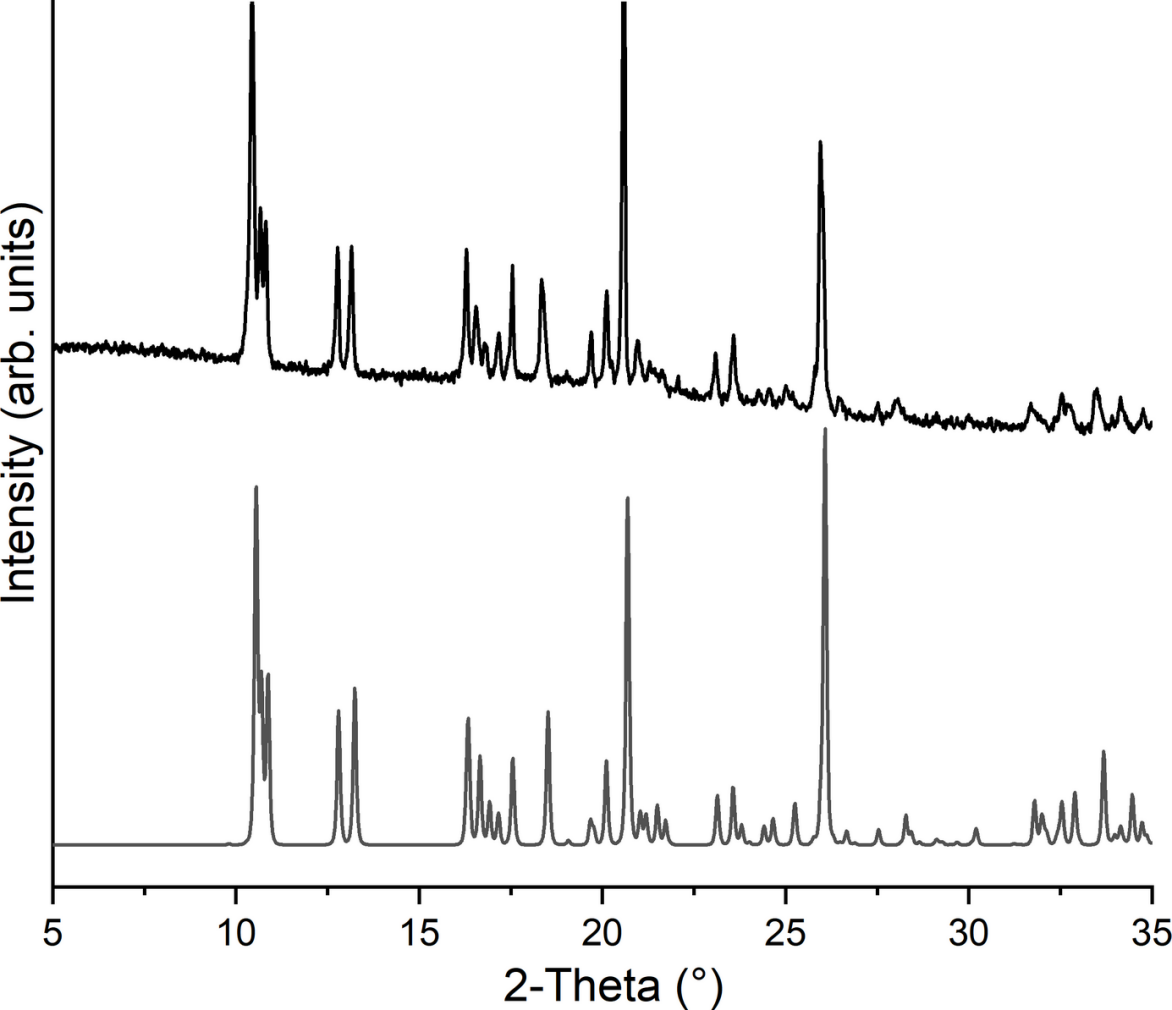
Experimental X-ray powder pattern of a sample of the title compound stored for 2 d in a humid atmosphere (top) and the calculated powder pattern (bottom).

Powder X-ray diffraction measurements prove that the title compound has been obtained as a pure phase (Fig. 4 ▸). In the IR and Raman spectrum the CN stretching vibration is observed at 2108 and 2095 cm^−1^ (IR) and at 2100 cm^−1^ (Raman), which confirms the presence of μ-1,3-bridging thio­cyanate anions (Fig. 5 ▸). To determine whether the title compound can be transformed into a discrete aqua complex with the composition Co(NCS)_2_(4-methyl­pyridine)_2_(H_2_O)_2_, which exists for the corresponding compound with 4-chloro­pyridine (Böhme *et al.*, 2020 ▸), a sample of the title compound was stored for 2 d in a humid atmosphere, but no changes were observed (Fig. 6 ▸).

## 6. Synthesis and crystallization

**Synthesis**

4-Methyl­pyridine and Co(NCS)_2_ were obtained from Sigma-Aldrich. The title compound was prepared by the reaction of Co(NCS)_2_ (350.2 mg, 2.06 mmol) and 4-methyl­pyridine (100 µL, 1.03 mmol) in 3 mL of water. The reaction mixture was stirred for 2 d at 393 K in a closed glass tube. C_14_H_14_CoN_4_S_2_ (361.34): calculated C 46.53, H 3.91, N 15.50, S 17.75; found C 46.2, H 3.7, N 15.3, S 17.4. Single crystals were prepared by the same method without stirring. The purity was proven by powder X-ray diffraction (see Fig. 4 ▸). An IR and a Raman spectrum of the title compound can be seen in Fig. 5 ▸.

**Experimental details**

Elemental analysis was performed with a vario MICRO cube from Elementar Analysensysteme GmbH. IR spectra were recorded at room temperature on a Bruker Vertex70 FT-IR spectrometer using a broadband spectral range extension VERTEX FM for full mid and far IR. Raman spectra were recorded on a Bruker RAM II FT-Raman spectrometer using a liquid nitro­gen cooled, highly sensitive Ge detector, 1064 nm radiation and 3 cm^−1^ resolution. X-ray powder diffraction experiments were performed using a Stoe STADI P transmission powder diffractometer with Cu *K*α_1_ radiation (λ = 1.540598 Å), a Johann-type Ge(111) monochromator and a MYTHEN 1K detector from Dectris.

## 7. Refinement

Crystal data, data collection and structure refinement details are summarized in Table 3 ▸. The hydrogen atoms were positioned with idealized geometry (methyl H atoms allowed to rotate but not to tip) and were refined with *U*_iso_(H) = 1.2*U*_eq_(C) (1.5 for methyl H atoms) using a riding model. The methyl H atoms in both crystallographically independent 4-methyl­pyridine ligands are disordered and were refined in two orientations rotated by 60°.

## Supplementary Material

Crystal structure: contains datablock(s) I. DOI: 10.1107/S2056989024012003/ox2010sup1.cif

Structure factors: contains datablock(s) I. DOI: 10.1107/S2056989024012003/ox2010Isup2.hkl

CCDC reference: 2409370

Additional supporting information:  crystallographic information; 3D view; checkCIF report

## Figures and Tables

**Table 1:** Selected geometric parameters (Å, °)

Co1—N1	2.0699 (16)	Co2—S1	2.5752 (4)
Co1—S2	2.6138 (6)	Co2—N2	2.0585 (16)
Co1—N11	2.1437 (16)	Co2—N21	2.1768 (16)
			
N1^i^—Co1—N1	174.16 (10)	N2—Co2—S1	93.94 (4)
N1^i^—Co1—S2	82.53 (5)	N2^ii^—Co2—S1	86.06 (5)
N1—Co1—S2	93.32 (5)	N2—Co2—N2^ii^	180.00 (10)
N1^i^—Co1—N11	93.35 (6)	N2—Co2—N21^ii^	90.54 (6)
N1—Co1—N11	90.80 (6)	N2—Co2—N21	89.46 (6)
S2^i^—Co1—S2	89.82 (3)	N21—Co2—S1	90.10 (4)
N11—Co1—S2	90.71 (4)	N21^ii^—Co2—S1^ii^	90.09 (4)
N11^i^—Co1—S2	173.33 (4)	N21^ii^—Co2—S1	89.90 (4)
N11—Co1—N11^i^	89.54 (8)	N21—Co2—N21^ii^	180.0
S1—Co2—S1^ii^	180.0		

Symmetry codes: (i) 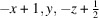; (ii) 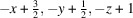.

**Table 2:** Hydrogen-bond geometry (Å, °)

*D*—H⋯*A*	*D*—H	H⋯*A*	*D*⋯*A*	*D*—H⋯*A*
C15—H15⋯N1	0.94	2.67	3.131 (3)	111

**Table 3:** Experimental details

Crystal data	
Chemical formula	[Co(NCS)_2_(C_6_H_7_N)_2_]
*M*_r_	361.34
Crystal system, space group	Monoclinic, *C*2/*c*
Temperature (K)	220
*a*, *b*, *c* (Å)	20.1106 (12), 9.2112 (4), 19.2309 (12)
β (°)	116.353 (6)
*V* (Å^3^)	3192.2 (3)
*Z*	8
Radiation type	Mo *K*α
μ (mm^−1^)	1.33
Crystal size (mm)	0.19 × 0.15 × 0.12

Data collection	
Diffractometer	Stoe *IPDS2*
Absorption correction	Numerical (*X-RED* and *X-SHAPE*; Stoe, 2008 ▸)
*T*_min_, *T*_max_	0.685, 0.763
No. of measured, independent and observed [*I* > 2σ(*I*)] reflections	18094, 3840, 3249
*R*_int_	0.030

Refinement	
*R*[*F*^2^ > 2σ(*F*^2^)], *wR*(*F*^2^), *S*	0.035, 0.095, 1.04
No. of reflections	3840
No. of parameters	195
H-atom treatment	H-atom parameters constrained
Δρ_max_, Δρ_min_ (e Å^−3^)	0.54, −0.44

Computer programs: *X-AREA* (Stoe, 2008 ▸), *SHELXT* (Sheldrick, 2015*a* ▸), *SHELXL* (Sheldrick, 2015*b* ▸), *DIAMOND* (Brandenburg & Putz, 1999 ▸), *XP* in *SHELXTL* (Sheldrick, 2008 ▸) and *publCIF* (Westrip, 2010 ▸).

## supplementary crystallographic information

### catena-Poly[[bis(4-methylpyridine-κN)cobalt(II)]-di-µ-thiocyanato-κ2N:S;κ2S:N] Crystal data

**Table:**

[Co(NCS)_2_(C_6_H_7_N)_2_]	*F*(000) = 1480
*M**_r_* = 361.34	*D*_x_ = 1.504 Mg m^−^^3^
Monoclinic, *C*2/*c*	Mo *K*α radiation, λ = 0.71073 Å
*a* = 20.1106 (12) Å	Cell parameters from 18094 reflections
*b* = 9.2112 (4) Å	θ = 2.4–28.0°
*c* = 19.2309 (12) Å	µ = 1.33 mm^−^^1^
β = 116.353 (6)°	*T* = 220 K
*V* = 3192.2 (3) Å^3^	Block, violet
*Z* = 8	0.19 × 0.15 × 0.12 mm

### catena-Poly[[bis(4-methylpyridine-κN)cobalt(II)]-di-µ-thiocyanato-κ2N:S;κ2S:N] Data collection

**Table:**

Stoe IPDS-2 diffractometer	3249 reflections with *I* > 2σ(*I*)
Graphite monochromator	*R*_int_ = 0.030
ω scans	θ_max_ = 28.0°, θ_min_ = 2.4°
Absorption correction: numerical (X-Red and X-Shape; Stoe, 2008)	*h* = −26→26
*T*_min_ = 0.685, *T*_max_ = 0.763	*k* = −12→12
18094 measured reflections	*l* = −25→25
3840 independent reflections	

### catena-Poly[[bis(4-methylpyridine-κN)cobalt(II)]-di-µ-thiocyanato-κ2N:S;κ2S:N] Refinement

**Table:**

Refinement on *F*^2^	Hydrogen site location: inferred from neighbouring sites
Least-squares matrix: full	H-atom parameters constrained
*R*[*F*^2^ > 2σ(*F*^2^)] = 0.035	*w* = 1/[σ^2^(*F*_o_^2^) + (0.0668*P*)^2^ + 0.3181*P*] where *P* = (*F*_o_^2^ + 2*F*_c_^2^)/3
*wR*(*F*^2^) = 0.095	(Δ/σ)_max_ = 0.001
*S* = 1.04	Δρ_max_ = 0.54 e Å^−^^3^
3840 reflections	Δρ_min_ = −0.44 e Å^−^^3^
195 parameters	Extinction correction: SHELXL-2016/6 (Sheldrick 2015b), Fc^*^=kFc[1+0.001xFc^2^λ^3^/sin(2θ)]^-1/4^
0 restraints	Extinction coefficient: 0.0057 (5)

### catena-Poly[[bis(4-methylpyridine-κN)cobalt(II)]-di-µ-thiocyanato-κ2N:S;κ2S:N] Special details

**Table:**

Geometry. All esds (except the esd in the dihedral angle between two l.s. planes) are estimated using the full covariance matrix. The cell esds are taken into account individually in the estimation of esds in distances, angles and torsion angles; correlations between esds in cell parameters are only used when they are defined by crystal symmetry. An approximate (isotropic) treatment of cell esds is used for estimating esds involving l.s. planes.

### catena-Poly[[bis(4-methylpyridine-κN)cobalt(II)]-di-µ-thiocyanato-κ2N:S;κ2S:N] Fractional atomic coordinates and isotropic or equivalent isotropic displacement parameters (Å^2^)

**Table:**

	*x*	*y*	*z*	*U*_iso_*/*U*_eq_	Occ. (<1)
Co1	0.500000	0.49107 (4)	0.250000	0.02383 (11)	
Co2	0.750000	0.250000	0.500000	0.02301 (11)	
S1	0.66193 (2)	0.42603 (6)	0.52245 (2)	0.02860 (13)	
C1	0.59987 (9)	0.4576 (2)	0.43277 (10)	0.0227 (3)	
N1	0.55673 (9)	0.47963 (18)	0.36976 (9)	0.0282 (3)	
S2	0.58146 (3)	0.29010 (6)	0.22966 (3)	0.03552 (15)	
C2	0.64772 (10)	0.2750 (2)	0.31844 (10)	0.0241 (3)	
N2	0.69410 (9)	0.26457 (19)	0.38096 (9)	0.0289 (3)	
N11	0.57409 (8)	0.65628 (18)	0.24774 (9)	0.0272 (3)	
C11	0.59645 (10)	0.6656 (2)	0.19205 (11)	0.0295 (4)	
H11	0.577925	0.597731	0.151360	0.035*	
C12	0.64542 (11)	0.7701 (2)	0.19119 (11)	0.0307 (4)	
H12	0.658799	0.772892	0.150181	0.037*	
C13	0.67484 (10)	0.8707 (2)	0.25094 (11)	0.0293 (4)	
C14	0.65122 (12)	0.8613 (2)	0.30850 (13)	0.0375 (5)	
H14	0.669143	0.927491	0.349996	0.045*	
C15	0.60160 (12)	0.7551 (2)	0.30501 (12)	0.0358 (5)	
H15	0.586236	0.751641	0.344562	0.043*	
C16	0.72997 (13)	0.9827 (3)	0.25418 (15)	0.0423 (5)	
H16A	0.719681	1.011652	0.201869	0.063*	0.3193
H16B	0.779623	0.942496	0.280015	0.063*	0.3193
H16C	0.726370	1.066719	0.282752	0.063*	0.3193
H16D	0.764102	1.002260	0.307889	0.063*	0.6807
H16E	0.704159	1.071415	0.229743	0.063*	0.6807
H16F	0.757413	0.947192	0.227005	0.063*	0.6807
N21	0.67949 (8)	0.06822 (18)	0.49669 (9)	0.0280 (3)	
C21	0.70667 (11)	−0.0423 (2)	0.54644 (12)	0.0348 (4)	
H21	0.758041	−0.045551	0.578635	0.042*	
C22	0.66266 (12)	−0.1523 (2)	0.55279 (13)	0.0374 (4)	
H22	0.684392	−0.227674	0.588788	0.045*	
C23	0.58650 (11)	−0.1515 (2)	0.50608 (12)	0.0306 (4)	
C24	0.55912 (11)	−0.0391 (2)	0.45241 (12)	0.0333 (4)	
H24	0.508301	−0.035451	0.417935	0.040*	
C25	0.60634 (10)	0.0673 (2)	0.44959 (11)	0.0304 (4)	
H25	0.586322	0.142359	0.413022	0.036*	
C26	0.53661 (13)	−0.2648 (2)	0.51418 (14)	0.0404 (5)	
H26A	0.485301	−0.234544	0.485588	0.061*	0.6344
H26B	0.548454	−0.276466	0.568562	0.061*	0.6344
H26C	0.543875	−0.356370	0.493626	0.061*	0.6344
H26D	0.566452	−0.343709	0.546263	0.061*	0.3656
H26E	0.503299	−0.301788	0.463289	0.061*	0.3656
H26F	0.507879	−0.221883	0.538224	0.061*	0.3656

### catena-Poly[[bis(4-methylpyridine-κN)cobalt(II)]-di-µ-thiocyanato-κ2N:S;κ2S:N] Atomic displacement parameters (Å^2^)

**Table:**

	*U*^11^	*U*^22^	*U*^33^	*U*^12^	*U*^13^	*U*^23^
Co1	0.01963 (17)	0.0287 (2)	0.01631 (17)	0.000	0.00182 (13)	0.000
Co2	0.01948 (17)	0.0281 (2)	0.01689 (17)	0.00358 (12)	0.00393 (13)	0.00223 (13)
S1	0.0267 (2)	0.0373 (3)	0.0161 (2)	0.00936 (18)	0.00425 (17)	0.00090 (17)
C1	0.0198 (7)	0.0243 (8)	0.0228 (8)	0.0022 (6)	0.0083 (6)	−0.0016 (7)
N1	0.0247 (7)	0.0350 (9)	0.0190 (7)	0.0046 (6)	0.0044 (6)	−0.0003 (6)
S2	0.0337 (3)	0.0424 (3)	0.0187 (2)	0.0092 (2)	0.00094 (19)	−0.00481 (19)
C2	0.0243 (8)	0.0255 (9)	0.0229 (8)	0.0033 (6)	0.0109 (7)	0.0013 (7)
N2	0.0274 (7)	0.0356 (9)	0.0202 (7)	0.0072 (6)	0.0075 (6)	0.0015 (6)
N11	0.0232 (7)	0.0311 (8)	0.0246 (7)	−0.0022 (6)	0.0082 (6)	−0.0051 (6)
C11	0.0295 (8)	0.0335 (10)	0.0230 (8)	−0.0015 (7)	0.0093 (7)	−0.0077 (8)
C12	0.0329 (9)	0.0344 (10)	0.0285 (9)	−0.0002 (8)	0.0170 (8)	−0.0036 (8)
C13	0.0252 (8)	0.0294 (10)	0.0328 (9)	−0.0006 (7)	0.0125 (7)	−0.0041 (8)
C14	0.0426 (11)	0.0395 (12)	0.0343 (10)	−0.0130 (9)	0.0205 (9)	−0.0160 (9)
C15	0.0428 (11)	0.0392 (11)	0.0317 (10)	−0.0107 (9)	0.0224 (9)	−0.0133 (9)
C16	0.0437 (12)	0.0404 (12)	0.0498 (13)	−0.0125 (9)	0.0271 (11)	−0.0095 (10)
N21	0.0245 (7)	0.0297 (9)	0.0272 (8)	0.0017 (6)	0.0092 (6)	0.0005 (6)
C21	0.0287 (9)	0.0331 (10)	0.0347 (10)	0.0013 (8)	0.0069 (8)	0.0033 (9)
C22	0.0380 (10)	0.0312 (11)	0.0363 (10)	0.0011 (8)	0.0106 (9)	0.0057 (9)
C23	0.0343 (9)	0.0289 (9)	0.0329 (9)	−0.0008 (8)	0.0187 (8)	−0.0064 (8)
C24	0.0256 (8)	0.0385 (11)	0.0350 (10)	0.0006 (8)	0.0126 (8)	−0.0046 (9)
C25	0.0260 (8)	0.0340 (10)	0.0288 (9)	0.0049 (7)	0.0101 (7)	0.0022 (8)
C26	0.0438 (11)	0.0358 (11)	0.0477 (12)	−0.0072 (9)	0.0260 (10)	−0.0082 (10)

### catena-Poly[[bis(4-methylpyridine-κN)cobalt(II)]-di-µ-thiocyanato-κ2N:S;κ2S:N] Geometric parameters (Å, º)

**Table:**

Co1—N1^i^	2.0699 (16)	C14—C15	1.377 (3)
Co1—N1	2.0699 (16)	C15—H15	0.9400
Co1—S2^i^	2.6138 (6)	C16—H16A	0.9700
Co1—S2	2.6138 (6)	C16—H16B	0.9700
Co1—N11	2.1437 (16)	C16—H16C	0.9700
Co1—N11^i^	2.1437 (16)	C16—H16D	0.9700
Co2—S1	2.5752 (4)	C16—H16E	0.9700
Co2—S1^ii^	2.5753 (4)	C16—H16F	0.9700
Co2—N2	2.0585 (16)	N21—C21	1.336 (3)
Co2—N2^ii^	2.0585 (16)	N21—C25	1.342 (2)
Co2—N21	2.1768 (16)	C21—H21	0.9400
Co2—N21^ii^	2.1768 (16)	C21—C22	1.386 (3)
S1—C1	1.6448 (18)	C22—H22	0.9400
C1—N1	1.153 (2)	C22—C23	1.390 (3)
S2—C2	1.6401 (18)	C23—C24	1.392 (3)
C2—N2	1.153 (2)	C23—C26	1.503 (3)
N11—C11	1.336 (2)	C24—H24	0.9400
N11—C15	1.344 (2)	C24—C25	1.382 (3)
C11—H11	0.9400	C25—H25	0.9400
C11—C12	1.382 (3)	C26—H26A	0.9700
C12—H12	0.9400	C26—H26B	0.9700
C12—C13	1.388 (3)	C26—H26C	0.9700
C13—C14	1.387 (3)	C26—H26D	0.9700
C13—C16	1.495 (3)	C26—H26E	0.9700
C14—H14	0.9400	C26—H26F	0.9700
			
N1^i^—Co1—N1	174.16 (10)	C13—C16—H16D	109.5
N1^i^—Co1—S2	82.53 (5)	C13—C16—H16E	109.5
N1—Co1—S2	93.32 (5)	C13—C16—H16F	109.5
N1^i^—Co1—S2^i^	93.32 (5)	H16A—C16—H16B	109.5
N1—Co1—S2^i^	82.53 (5)	H16A—C16—H16C	109.5
N1^i^—Co1—N11	93.35 (6)	H16A—C16—H16D	141.1
N1^i^—Co1—N11^i^	90.80 (6)	H16A—C16—H16E	56.3
N1—Co1—N11	90.80 (6)	H16A—C16—H16F	56.3
N1—Co1—N11^i^	93.34 (6)	H16B—C16—H16C	109.5
S2^i^—Co1—S2	89.82 (3)	H16B—C16—H16D	56.3
N11—Co1—S2	90.71 (4)	H16B—C16—H16E	141.1
N11^i^—Co1—S2	173.33 (4)	H16B—C16—H16F	56.3
N11—Co1—S2^i^	173.33 (4)	H16C—C16—H16D	56.3
N11^i^—Co1—S2^i^	90.71 (4)	H16C—C16—H16E	56.3
N11—Co1—N11^i^	89.54 (8)	H16C—C16—H16F	141.1
S1—Co2—S1^ii^	180.0	H16D—C16—H16E	109.5
N2—Co2—S1	93.94 (4)	H16D—C16—H16F	109.5
N2^ii^—Co2—S1^ii^	93.94 (4)	H16E—C16—H16F	109.5
N2^ii^—Co2—S1	86.06 (5)	C21—N21—Co2	120.72 (13)
N2—Co2—S1^ii^	86.06 (4)	C21—N21—C25	117.11 (18)
N2—Co2—N2^ii^	180.00 (10)	C25—N21—Co2	121.95 (14)
N2—Co2—N21^ii^	90.54 (6)	N21—C21—H21	118.5
N2—Co2—N21	89.46 (6)	N21—C21—C22	123.07 (19)
N2^ii^—Co2—N21	90.54 (6)	C22—C21—H21	118.5
N2^ii^—Co2—N21^ii^	89.46 (7)	C21—C22—H22	119.9
N21—Co2—S1	90.10 (4)	C21—C22—C23	120.2 (2)
N21^ii^—Co2—S1^ii^	90.09 (4)	C23—C22—H22	119.9
N21^ii^—Co2—S1	89.90 (4)	C22—C23—C24	116.29 (18)
N21—Co2—S1^ii^	89.91 (4)	C22—C23—C26	121.6 (2)
N21—Co2—N21^ii^	180.0	C24—C23—C26	122.07 (19)
C1—S1—Co2	101.18 (6)	C23—C24—H24	119.9
N1—C1—S1	179.55 (16)	C25—C24—C23	120.27 (18)
C1—N1—Co1	164.70 (14)	C25—C24—H24	119.9
C2—S2—Co1	100.25 (6)	N21—C25—C24	122.98 (19)
N2—C2—S2	179.70 (18)	N21—C25—H25	118.5
C2—N2—Co2	162.72 (14)	C24—C25—H25	118.5
C11—N11—Co1	123.13 (13)	C23—C26—H26A	109.5
C11—N11—C15	116.78 (17)	C23—C26—H26B	109.5
C15—N11—Co1	120.08 (13)	C23—C26—H26C	109.5
N11—C11—H11	118.3	C23—C26—H26D	109.5
N11—C11—C12	123.36 (17)	C23—C26—H26E	109.5
C12—C11—H11	118.3	C23—C26—H26F	109.5
C11—C12—H12	120.0	H26A—C26—H26B	109.5
C11—C12—C13	119.95 (17)	H26A—C26—H26C	109.5
C13—C12—H12	120.0	H26A—C26—H26D	141.1
C12—C13—C16	122.08 (17)	H26A—C26—H26E	56.3
C14—C13—C12	116.57 (18)	H26A—C26—H26F	56.3
C14—C13—C16	121.35 (18)	H26B—C26—H26C	109.5
C13—C14—H14	119.9	H26B—C26—H26D	56.3
C15—C14—C13	120.20 (18)	H26B—C26—H26E	141.1
C15—C14—H14	119.9	H26B—C26—H26F	56.3
N11—C15—C14	123.12 (17)	H26C—C26—H26D	56.3
N11—C15—H15	118.4	H26C—C26—H26E	56.3
C14—C15—H15	118.4	H26C—C26—H26F	141.1
C13—C16—H16A	109.5	H26D—C26—H26E	109.5
C13—C16—H16B	109.5	H26D—C26—H26F	109.5
C13—C16—H16C	109.5	H26E—C26—H26F	109.5
			
Co1—N11—C11—C12	−178.58 (15)	C15—N11—C11—C12	0.0 (3)
Co1—N11—C15—C14	177.88 (18)	C16—C13—C14—C15	−178.6 (2)
Co2—N21—C21—C22	172.50 (17)	N21—C21—C22—C23	0.2 (3)
Co2—N21—C25—C24	−172.73 (15)	C21—N21—C25—C24	2.0 (3)
N11—C11—C12—C13	1.0 (3)	C21—C22—C23—C24	2.2 (3)
C11—N11—C15—C14	−0.8 (3)	C21—C22—C23—C26	−176.8 (2)
C11—C12—C13—C14	−1.3 (3)	C22—C23—C24—C25	−2.5 (3)
C11—C12—C13—C16	177.9 (2)	C23—C24—C25—N21	0.4 (3)
C12—C13—C14—C15	0.6 (3)	C25—N21—C21—C22	−2.3 (3)
C13—C14—C15—N11	0.5 (4)	C26—C23—C24—C25	176.55 (18)

Symmetry codes: (i) −*x*+1, *y*, −*z*+1/2; (ii) −*x*+3/2, −*y*+1/2, −*z*+1.

### catena-Poly[[bis(4-methylpyridine-κN)cobalt(II)]-di-µ-thiocyanato-κ2N:S;κ2S:N] Hydrogen-bond geometry (Å, º)

**Table:**

*D*—H···*A*	*D*—H	H···*A*	*D*···*A*	*D*—H···*A*
C15—H15···N1	0.94	2.67	3.131 (3)	111
